# Outdoor Environment and Pediatric Asthma: An Update on the Evidence from North America

**DOI:** 10.1155/2017/8921917

**Published:** 2017-01-23

**Authors:** Jenna Pollock, Lu Shi, Ronald W. Gimbel

**Affiliations:** Department of Public Health Sciences, Clemson University, Clemson, SC, USA

## Abstract

*Introduction.* The evidence about the association between asthma and outdoor environmental factors has been inadequate for certain allergens. Even less is known about how these associations vary across seasons and climate regions. We reviewed recent literature from North America for research related to outdoor environmental factors and pediatric asthma, with attention to spatial-temporal variations of these associations.* Method.* We included indexed literature between years 2010 and 2015 on outdoor environmental factors and pediatric asthma, by searching PubMed.* Results.* Our search resulted in 33 manuscripts. Studies about the link between pediatric asthma and traffic-related air pollutants (TRAP) consistently confirmed the correlation between TRAP and asthma. For general air pollution, the roles of PM_2.5_ and CO were consistent across studies. The link between asthma and O_3_ varied across seasons. Regional variation exists in the role of SO_2_. The impact of pollen was consistent across seasons, whereas the role of polycyclic aromatic hydrocarbon was less consistent.* Discussion.* Recent studies strengthened the evidence about the roles of PM_2.5_, TRAP, CO, and pollen in asthma, while the evidence for roles of PM_10-2.5_, PM_10_, O_3_, NO_2_, SO_2_, and polycyclic aromatic hydrocarbon in asthma was less consistent. Spatial-temporal details of the environment are needed in future studies of asthma and environment.

## 1. Introduction

A scientific and political discourse on climate change and its impact on human health has captured substantial attention during the past decade [1–10]. In the United States, a chief driver behind the contemporary interest in climate change and human health is arguably the formal reports issued from The White House and federal agencies (e.g., National Institutes of Health, Environmental Protection Agency) [11–13]. These reports raise an alarm to increasing ground-level ozone levels, increasing pollution such as particulate matter (PM), carbon monoxide (CO), nitrogen dioxide (NO_2_) through wildfires, extreme heat events, and higher aeroallergen concentrations, all of which may impact the health of at-risk populations, especially children suffering from asthma, respiratory allergies, and airway disease [12]. Academia and medical societies have also contributed to the ongoing discussion by outlining the threats of climate change on respiratory diseases and related illness, specifically highlighting the burden on children [14, 15].

Much of the concern on climate change is about the influence of a changing outdoor environment in relation to children living with asthma and respiratory diseases. Asthma, compounded with the common comorbid condition of allergic rhinitis, is one of the most prevalent chronic diseases among children worldwide [16, 17]. Children have immature lungs and airways which are more susceptible to inflammation than their adult counterparts; children between 6 and 18 years, as compared with adults, are at high risk for emergency hospitalization for asthma [18]. Asthma continues to pose a substantial burden to child and adult health, with approximately 9.6% of children in the United States living with the disease [19, 20]. Asthma appears to disproportionately affect children from minority and impoverished communities [19, 21]. A review of the scientific literature, covering periods of 2006–2009, explored the relationship between outdoor air pollution and asthma in children [22]. This 2011 review evaluated a total of 25 articles found using the key search words “outdoor air pollution, asthma, and children” in PubMed and included children ranging from birth to 18 years of age [22]. Findings from this review found general associations between chronic ozone, nitrogen dioxide (NO_2_), particular matters (PMs), wood smoke, and traffic exhaust exposure and exacerbation of asthma symptoms across the board. Suggestions for future studies included directing research towards specific pollutants for a more informative association of outdoor exposures and asthma exacerbation. The evidence that suggests an established associated link between outdoor air pollution and pediatric asthma exacerbation is compelling [22, 23]. However, without confirmed link between specific aeroallergens and asthma outcomes under specific weather settings, it remains difficult to infer the associated relation between climate change and changes in asthma prevalence. The suggested associated pathway between the recent warming by latitude and the longer ragweed-pollen season in North America might be a plausible hypothesis to explain higher prevalence of pediatric asthma [24], yet whether this longer ragweed-pollen season has led to more asthma attacks remains untested. With the advancement in technology [25] and data availability [26], however, the research community has been able to examine more specific association between outdoor aeroallergens and health outcomes [27, 28] under different spatial-temporal settings. A narrative review that updates the recent literature is necessary to summarize the recent findings about outdoor environment and pediatric asthma.

The authors aim to review the recent scientific literature related to outdoor environment and pediatric asthma by (1) providing a narrative review of the recent literature studying associations between factors in the outdoor environment and pediatric asthma, with special attention to the possible spatial-temporal variations of these associations, (2) identifying possible gaps between current research and the impact of climate change on asthma in children outlined in federal reports, and (3) providing recommendations on how the use of health information technology and “big data” might enhance current and future research.

## 2. Methods

We conducted our review by searching the PubMed search engine. We included all indexed scientific literature, published between years 2010 and 2015, on outdoor environmental factors and asthma in children. We incorporated the following MeSH terms and condition in our search:

((“Asthma/chemically induced” [Mesh] OR “Asthma/epidemiology” [Mesh] OR “Asthma/etiology” [Mesh] OR “Asthma/immunology” [Mesh] OR “Asthma/pathology” [Mesh] OR “Asthma/physiology” [Mesh] OR “Asthma/prevention and control” [Mesh]) AND (“humans” [MeSH Terms] AND English [lang])) AND (environmental factors [TI] OR (“Environmental Exposure” [Mesh] OR “Environmental Illness” [Mesh])) AND “2010/02/26” [PDat]: “2015/02/24” [PDat].

To ensure rigor we developed a review protocol that two of our three authors (Jenna Pollock and Ronald W. Gimbel) followed in the review process. The protocol included our search terms and inclusion/exclusion criteria.

### 2.1. Inclusion and Exclusion Criteria

Studies were included if they met the following criteria: (1) research conducted in North America, (2) research participants or focus was on children (<18 years of age), (3) manuscripts incorporated a scientific collection and analysis of data to include, but not limited to, randomized controlled trials, cross-sectional data analysis case controls, cohort prospective studies, epidemiological studies literature reviews (systematic and narrative), or cohort retrospective trials, and (4) published between 2010–2015. Studies were excluded if (1) manuscript were not considered research (e.g., opinion papers) and (2) manuscripts were not written in English.

### 2.2. Review Process

Two authors (Jenna Pollock and Ronald W. Gimbel) incorporated a three-step review process that included an initial title review, abstract review, and full-text review. Both authors conducted independent reviews and compared findings. When a finding differed between authors, each collectively reviewed points of differentiation and a consensus vote was achieved. We eliminated duplicate titles and titles that did not meet the inclusion criteria or met exclusion criteria. Then we repeated this procedure in our abstract review and full-text review.

## 3. Results

**Figure 1:**
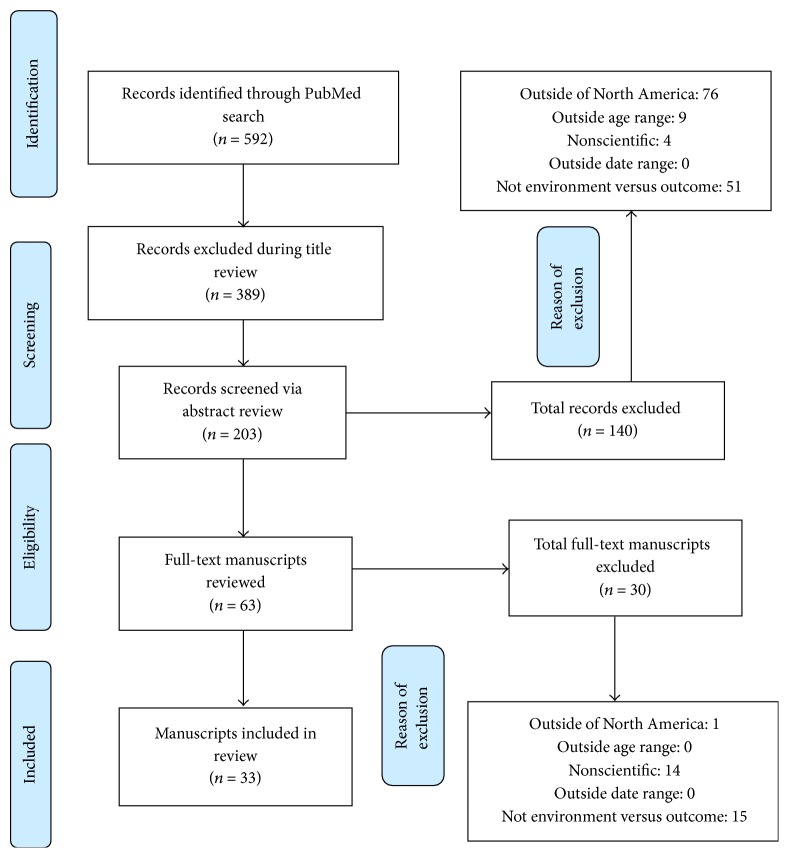
Flow diagram of our manuscript selection.

Our literature search yielded a total of 530 manuscripts with 356 being excluded in title review. The remaining 174 manuscripts were assessed through abstract review; 128 were excluded at this stage. The final full-text review of 46 manuscripts resulted in the exclusion of 13 manuscripts, leaving 33 manuscripts included in this manuscript (Figure 1). Tables 1–3 identify each paper reviewed and includes environmental variables studied, age group, sample size, climate region, and any methods used during the study. Given the complexity and heterogeneity in defining asthma outcomes, we stratify these studies into four categories in our tables (Tables 1–3): asthma diagnosis and symptoms, asthma symptoms, asthma symptom and care utilization, and asthma cost. These three tables include both the findings and the limits of each included study. These included manuscripts are identified in three focused areas below, followed by a review of identified literature review (narrative and systematic) and intervention studies.

### 3.1. Research Focused Primarily on General Traffic-Related Air Pollution “TRAP” (Table 1)

Traffic-related air pollution is a collective concept that includes the various pollutants associated with traffic. It has been challenging to determine the impact of the specific types of pollutants on respiratory function [29]. Table 1 summarizes the findings about child asthma and the aggregate measure of traffic-related air pollution (TRAP). Other studies measured impacts of specific pollutants of TRAP such as carbon monoxide, ultrafine particles, ozone, nitrogen dioxide, black carbon, sulfide dioxide, and other lesser-mentioned pollutants on asthma outcomes [30]. Because these studies examined specific pollutants associated with traffic-related air pollution, the associations between these pollutants and pediatric asthma were reported separately in Table 2.

Five studies focused on generalized TRAP as a whole unit rather than specific TRAP markers as described above. While the methods and types of asthmatic markers measured differ between the studies, the results from McConnell et al., Bernstein, Eckel et al., Newman et al., and Sucharew et al. [29, 31–34] consistently uphold what has been shown in earlier work regarding the link between TRAP and asthma. Of the studies adding evidence to the association between TRAP and asthma, McConnell et al. [29] found that asthma risk increased with modeled traffic-related pollution exposure from roadways near homes [Hazard Ratio (HR): 1.51; 95% confidence interval (CI), 1.25–1.82] and near schools [HR 1.45; 95% CI, 1.06–1.98]. Bernstein [31] found that one's exposure to “stop and go” traffic was associated with wheezing during infancy. Eckel et al. [32] found that the length of road was positively associated with fractional exhaled nitric oxide (FeNO) among pediatric patients, a marker commonly used to measure oxidative stress and airway inflammation. Sucharew et al. (2010) found that children exposed to the highest tertile of traffic exhaust had an estimated 45% increase in risk of recurrent night cough (RNC), compared with children exposed to the lowest tertile [adjusted Odds Ratio 1.45, 95% CI: 1.09, 1.94]. Finally, we did identify one study that did not report a significant association between TRAP exposure and asthma, while Newman et al. [33] reported that higher TRAP exposure was associated with a higher hospital readmission rate (21% versus 16%; *P* = 0.05); this association was not significant after adjusting for covariates [OR, 1.4; 95% CI, 0.9–2.2]. It may be noteworthy that this 2013 paper was the only study that explored hospital readmission rates as the marker for asthma outcome, which could be a function of treatment and management besides environmental triggers. The study's relatively small sample (758 children, 19% of whom had a hospital readmission) might also explain its lack of statistical significance after controlling for the covariates.

### 3.2. Research on Specific Air Pollutants (Table 2)

#### 3.2.1. Particulate Matter (PM)

Studies about particular matter and asthma among children examined the impact from three major types: PM_2.5_, PM_10-2.5_, and PM_10_. Eleven studies concluded that PM_2.5_ was associated with worsening asthma symptoms and/or increased oxidative stress as determined by biomarkers in pediatric patients [35–46]. For instance, a study by Delfino et al. [37] of 11,390 asthma-related hospital encounters among 7492 subjects aged 0–18 found significant and positive associations between PM_2.5_ and child asthma regardless of outdoor temperature (e.g., warm or cold season). On the other hand, Evans et al. and Patel et al. [30, 47] found neither significant nor positive association between asthma and PM_2.5_. For instance, Patel et al. [47] found no significant link between PM_2.5_ exposure and asthma in children in their 2013 paper, the only study that has studied 8-isoprostane as the asthma biomarker among our reviewed studies.

While PM_2.5_ is made up of several different components, among all our reviewed studies the one by Strickland et al. in 2010 [41] was the only team that made an association between asthma and PM_2.5_ components, including PM_2.5_ sulfate, PM_2.5_ elemental carbon, PM_2.5_ organic carbon, and PM_2.5_ water soluble metals within one location. Five papers examined the association between PM_10-2.5_ and asthma with different results [39, 41, 42, 45, 47]. Out of the five review papers, Lewis et al. and Patel et al. [39, 47] found no association; Patel et al. [47] found nonsignificant but positive association, while Strickland et al. and Sarnat et al. [41, 45] found positive and significant association. Among the seven studies specifically investigating PM_10_ and asthma in children there were also mixed results [35, 36, 39, 40, 42, 47, 48], with Berhane et al., Nishimura et al., and Patel et al. reporting no association [36, 40, 47], Akinbami et al., Lewis et al., and Lemke et al. reporting a significant and positive association [35, 39, 48], and Zora et al. reporting a nonsignificant but positive correlation [42].

In summary, there was a positive association, established in most (eleven out of thirteen) studies, between PM_2.5_ and asthma in children. On the other hand, research on the association between PM_10-2.5_ and PM_10_ with asthma in children recorded inconsistent findings. These results may be inconsistent due to failure to stratify between particulate matter subtypes, length of symptom tracking (ranging from 4 weeks to 11 years), or differences in the measurement of asthma severity. Some of these studies used a questionnaire to determine quality of asthma severity, while others used FeNO concentrations, 8-isoprostane concentrations, or number of emergency department visits to determine severity of asthma may be leading to variation of outcomes observed for larger PM sizes.

#### 3.2.2. Ozone (O_3_)

Children's exposure to ozone was a commonly studied risk factor and was measured to explore its associations with asthma outcomes in 10 of the 25 studies [30, 35–41, 49, 50]. Chronic exposure to ozone was found to increase asthma outcomes in studies by Akinbami et al., Lewis et al., Nishimura et al., and Perez et al. [35, 39, 40, 50] and increase costs in pediatric asthma outcomes in a study by Brandt et al. [49]. While the majority of evidence pointed towards a positive association between pediatric asthma and ozone exposure, there was little agreement as to the seasonality pattern in the association between ozone exposure and pediatric asthma. In Orange County of California, Delfino et al.'s study [37] of 11,390 asthma-related hospital encounters among 7492 subjects aged 0–18 found that asthma-related emergency department (ED) admissions was positively associated with ozone during the warm season, but not during the cool season. Meanwhile, Habre et al.'s longitudinal study [38] of 36 children found exposure was significantly associated with severe wheezing, especially during Fall and Spring. In Strickland et al.'s study [41] where daily counts of emergency department visits for asthma or wheeze among children aged 5–17 during 1993–2004 were collected from hospitals in Metropolitan Atlanta area, these ED visits were associated with ozone in both cold and warm seasons. In contrast to all these findings, Evans et al. [30] actually found a negative association between increased ozone concentration and asthma exacerbation among urban children, which the authors attributed to the negative correlation of ozone with nitrogen dioxide at the site of the study. Finally, Berhane et al. [36] found that ozone was not statistically significantly associated with FeNO asthma biomarker, yet this study did not take into account the season of measurement.

#### 3.2.3. Nitrogen Dioxide (NO_2_)

Eleven studies examined associations of NO_2_ and asthma [35–37, 40, 42, 44, 47–51]. The one study by Delfino et al. investigating NO_2_ by season found that during the cold season peaks in asthma cases were correlated [37] with NO_2_. Overall, of 11 papers which examined the association between NO_2_ and asthma, 10 found statistically significant and positive associations [35–37, 40, 42, 44, 47–51] and Akinbami et al. found a nonsignificant association [35]. In Nishimura et al.'s study [40] that took account of regional variations, there was association between NO_2_ and subsequent asthma events within 5 different urban regions in mainland United States and Puerto Rico. The one study by Akinbami et al. [35] finding no significant association studied 12-month NO_2_ levels in relation to the self-reported asthma outcomes in the National Health Interview Survey.

#### 3.2.4. Carbon Monoxide (CO) and Sulfur Dioxide (SO_2_)

Six studies studied SO_2_ [30, 35, 40, 41, 43, 48], among which Nishimura et al.'s study found that the significance of associations between SO_2_ and asthma varied among regions in the United States [40]. Evans et al., Akinbami et al., Spira-Cohen et al., and Lemke et al. found no significant association between SO_2_ and asthma [30, 35, 43, 48]. Strickland et al. [41] found SO_2_ to be associated during the warm season only. Meanwhile, the evidence about the link between carbon monoxide and asthma is much more consistent: all four papers (by Evans et al., Delfino et al., Strickland et al., and Vette et al.) [30, 37, 41, 44] that studied CO's impact found associations with asthma.

#### 3.2.5. Other Variables

Possible impacts from polycyclic aromatic hydrocarbons were explored in 4 studies: Lemke et al., Jung et al., Miller et al., and Padula et al. [48, 52–54], among which only the study by Lemke et al. [48] found positive, statistically significant impact from benzene and toluene (independently) on pediatric asthma. The other three studies all confirmed significant positive associations between pediatric asthma and polycyclic aromatic hydrocarbons [48, 52, 53]. Ratnapradipa et al. [55] found that children exposed to “wood, oil, smoke, soot, or exhaust” were at higher risk for early asthma diagnosis. A study by Tse et al. [56] on wildfires and asthma found that exposure to catastrophic wildfire smoke was associated with worsening asthma outcomes particularly in obese children. In a study of patients with atopy and a history of wheezing by Jerschow [57], asthma morbidity is associated with high urinary dichlorophenol levels, suggesting that plant pesticide with dichlorophenol might be another symptom trigger for asthma patients. Finally, the outdoor fungal exposure was found to be associated with increased asthma symptoms and increased risk of exacerbations according to a 2010 study of inner-city children by Pongracic et al. [58], adding to the literature about the possible link between fungal spore and asthma severity [59].

As urbanization may affect health through certain environmental exposures that may be more prevalent in urban environments (e.g., traffic pollution, industrial emissions, and noise), urban land use was found by Ebisu et al. [51] to significantly increase the wheezing severity among infants; the effects were mostly associated with TRAP rather than noise or stress though the latter two still played a minor factor in wheezing severity.

### 3.3. Research on Aeroallergens and Other Exposures (Table 3)

#### 3.3.1. Plant-Related Aeroallergens

Three manuscripts by Dellavalle et al., Jariwala et al., and Sheehan et al. [60–62] focused on the exposure to plant-based aeroallergens and asthma among children (Table 3), and all three studies identified strong correlations between tree pollen (even at low levels) and pediatric asthma, especially during the spring seasons. The correlation between tree pollen count and asthma-related emergency department visit peak in an urban area was not significant during the fall and winter season, as found by Jariwala et al. [61]. Grass and ragweed were the least common sensitizers in younger children (0–4 years), yet these aeroallergens became more prevalent in the older age groups (10–14 years), as found by Sheehan et al. [62].

#### 3.3.2. Effect of Race/Ethnicity and Socioeconomic Status on Outdoor Exposures and Asthma

It is possible that sociodemographic factors such as race/ethnicity and socioeconomic status play a confounding role between outdoor environment and asthma outcomes. Three studies by McConnell et al., Nishimura et al., and Ratnapradipa et al. [29, 40, 55] examined the role of race on the outcomes of various environmental variables on asthma. A study by Newman et al. on the readmission rates [33] found Caucasian children had a 3 times higher rate of readmission with high TRAP while African American children had no increased rate. Meanwhile, a 2013 study by Ratnapradipa et al. [55] showed a 31% increase in asthma prevalence among African American children related to “wood or oil smoke, soot, or exhaust.” According to the study by McConnell et al., [29] Hispanic children had the lowest rates of TRAP-based asthma and African American children the highest based on a small population of 1437 kindergarten students and first-graders in Southern California. One study studied the impact of environmental factors on the asthma outcome among minority children [40], but not at the differences across races/ethnicities and socioeconomic statuses [40]. No studies specifically explored the possible variation of the association between environmental factors and asthma outcomes across socioeconomic statuses.

### 3.4. Literature Reviews and Implementation Studies

A literature review by McGwin Jr. et al. [63] found evidence among seven studies linking formaldehyde exposure to worsening asthma in children but cited a need for further epidemiological studies on this topic to find conclusive evidence. A 2011 review by Tzivian about asthma and pollution between 2006 and 2009 confirmed a link between pollutants and asthma exacerbation but found variations of this link between the different age groups studied and discussed limitations in the measurement of outdoor pollutants [22].

Among studies with a theme of interventions and implementation, one study by YoussefAgha et al. [64] about the feasibility of environmental monitoring examined the integration of daily environmental health surveillance as a tool in predicting when best to apply precautionary measures for children. Focusing on the temporal pattern of asthma exacerbation, the authors found that the prior day CO, SO_2_, NO_2_, nitrogen monoxide (NO), PM_2.5_, and O_3_ had significant effects on asthma exacerbations among elementary school students in Pennsylvania, and they concluded that monitoring of air pollutants over time could be a reliable new means for predicting asthma exacerbations. With special attention to spatial variation of asthma triggers, an ongoing study by Vette et al. [44] explored new ways of measuring urban air pollutants by integrating measurement and modeling to quantify contribution of traffic sources to predict where pollutants would pose the greatest risk to children. More ambitiously, Gallagher et al.'s [65] ongoing study among Detroit children incorporated exposure metrics and clinical indicators to decipher the biological complexity with etiology related to gene-environment interactions, aiming to provide an opportunity to evaluate complex relationships between environmental factors, physiological biomarkers, genetic susceptibility, and asthma/cardiovascular outcomes.

Finally, while most of the discussion about intervention has been centered on environmental monitoring, Perez et al. pointed out that [50] encouraging compact growth in urban planning has also been suggested as an intersectoral approach, which would reduce pollution by making long vehicle travel less necessary.

## 4. Discussion

This review found 33 studies between 2010 and 2015 on outdoor environmental impacts on pediatric asthma. These studies have strengthened the evidence about the roles of PM2.5, TRAP, CO, and pollen in pediatric asthma, while the evidence for roles of PM_10-2.5_, PM_10_, O_3_, NO_2_, SO_2_, and polycyclic aromatic hydrocarbon in asthma has been less consistent. The link between an outdoor environment and childhood asthma has not been adequately examined with regard to the regional and temporal variation of the environment. Thus, spatial-temporal details of the environment are needed in future studies of asthma and environment, particularly if researchers want to examine the hypothesized impact of climate change on asthma. It is worth noting that some of the null results from our reviewed studies might be a result of study design rather than a lack of etiological link. For instance, Brandt et al. and Perez et al. [49, 50] found that risk assessment focusing on the effects of regional pollutants may underestimate the impact and the burden of air pollution due to challenges such as measurement and model specification.

One notable contribution of the recent studies about CO by Evans et al., Delfino et al., Strickland et al., and Vette et al. [30, 37, 41, 44] is the consistent association between carbon monoxide and asthma symptoms. While particular matter and ozone have been used for monitoring air quality as related to asthma outcome, carbon monoxide has been less often monitored and reported. It might be worthwhile to enhance the monitoring and reporting of carbon monoxide density as an air quality measure, especially for geographic areas where its density is often elevated.

There was a lack of studies regarding early childhood development and asthma. Asthma is difficult to diagnose in early childhood [66], an age when outdoor environment could pose a more serious threat to the exacerbation. Standardized research over age groups plus the adoption of diverse diagnostic tools for early childhood asthma will help clarify the differences between age groups as well as the exact role of pollution in asthma development.

In each of our three tables, the geographic location was specified to the distinct climate regions as defined by the National Centers for Environmental Information. These specifications protect against broad generalizations about environmental impacts on asthma, while limiting a study's relevance to the population's climate region. Only one study, the one by Nishimura et al. [40], explored differences between climate regions finding differences across 5 urban regions for the examined pollutant. So far, the geographic variation of pediatric asthma prevalence has been documented by Malhotra et al. [67] yet not fully understood. Future studies that explore the possible variation between asthma trends in different climate regions would help us understand better the possible role climate change might have played in asthma prevalence (via worsening ambient air pollution and altered local and regional pollen production, according to Shea et al. [68], which varies across different climate regions).

Only one study in our review, the one by Strickland et al. [41], broke down fine PM into its components. Reviewing the impact of the different components of particulate matter may help shape policy and research in the future pertaining to PM-induced asthma exacerbation. Compared with studies about PM and pediatric asthma, there are an insufficient number of studies exploring non-PM factors such as variations in temperature, seasonal environmental impact, pollen/weed levels, socioeconomic status, and race/ethnicity. Finding the precise impact of temperature on pediatric asthma is especially relevant as increasing bodies of evidence show rising temperatures across the globe [23], a significant proportion of which is based on human activities. Vette et al., YoussefAgha et al., and Gallagher et al. [44, 64, 65] discussed new ways to measure urban air pollution and design preventative plan. More research on these solutions will help to fine-tune and optimize policy actions addressing these outdoor environmental triggers of child asthma.

### 4.1. Gap between Federal Reports and Current Literature

There exists a research gap between the above-mentioned federal reports about climate changes' possible impact on respiratory diseases and the current literature as we reviewed; the link between outdoor environment and child asthma has not been adequately studied with regard to the regional and temporal variation of the environment. There is virtually no study about the exact temperature in the pediatric patient's location and asthma-related events. Without these spatial-temporal details about the outdoor environment for the study population, it is difficult to empirically examine the impact of climate change on child asthma outcome, even though the hypotheses about these associated pathways (wild fires, change in aeroallergen density, mold, insect population increase, etc.) are plausible. For instance, one climate-related factor, thunderstorm, has been shown as a significant trigger of asthma in England and Iran [69, 70], augmenting the previous findings about thunderstorm asthma [71]. Yet, after one 2008 paper from Atlanta, Georgia by Grundstein et al. [72], there has been little (if any) recent empirical study updating the evidence about thunderstorm and asthma in the United States and Canada.

### 4.2. Recommendations to Enhance Future Research Efforts

The increasingly affordable computational resources and the diversity of mobile health devices in recent years have enabled researchers to collect real-time health data from the very location where medical events take place. For example, obtaining the exact time and location of an asthma attack via mobile devices has become feasible [73] given the current stage of telecommunication technology, opening the door to constructing an “asthma registry” [74–76] where pieces of user-supplied geospatial and temporal input about asthma episodes are entered for surveillance and analysis. Piloting these kinds of data collections in places with high asthma prevalence could be the next feasible step in monitoring the impact of changing outdoor environment on child asthma.

## 5. Conclusion

Consistent with those studies documented prior to 2010, findings from this review of studies between 2010 and 2015 show that the associations between traffic-related air pollutants (including NO_2_, particulate matter, and SO_2_) and pediatric asthma are well supported. Seasonal and regional variations in certain outdoor factors were rarely accounted for, even though the studies that did study this found the variation to be significant. Future documentation about the specific composition of particulate matter and polycyclic aromatic hydrocarbons would add to studies that did not have the resources available to examine the underlying components of these environmental triggers. A surveillance system with standardized reporting of environmental effects across different age groups and geographic regions will clarify gaps in current knowledge on environmental impacts on pediatric asthma. New studies expanding the spatial dimension and temporal dimension of monitoring ambient levels of known environmental triggers and exacerbating agents are promising in the goal to prevent pediatric asthma ED visits and hospitalizations, enriching the toolkit for parents and community health stakeholders.

## Figures and Tables

**Table 1:** Studies focused on traffic-related air pollution (TRAP).

Source/year	Outdoor variables	Age group	Sample size	Climate region	Study design	Assessment method	Findings and limits
*Asthma symptom*							
Bernstein, 2012	TRAP (ECAT)	1–7 years	700	Central, US	Cohort/adjusted	Medical evaluations, skin testing, proximity, and LUR modeling^a^	Higher TRAP associated with wheezing during infancy and at age 3. Limit: parental reports of wheezing at 3 are not strong asthma predictors

McConnell et al., 2010	TRAP, PM_10_, PPM_2.5_, O_3_	K-1st grade	2,497	Western, US	Cohort/adjusted	Baseline and annual questionnaires, community ambient air pollution, weather variables, local TRAP	Asthma risk increased with modeled TRAP exposure^b^ from roadway near home (HR 1.51; 95% CI: 1.25–1.82) and near school (HR 1.45; 95% CI: 1.06–1.98). Limit: short 3-year follow-up

Sucharew et al., 2010	TRAP, PM_10_, PM_2.5_,	1–3 years	550	Central, US	Cohort/adjusted	Questionnaires, skin prick test, air quality monitoring, clinical evaluation, home visits, house dust	Children exposed to higher levels of TRAP are more likely to suffer recurrent night cough (OR, 1.45, 95% CI, 1.09–1.94) than children less exposed. Limit: sample is limited to those with high risk

Eckel et al., 2011	TRAP, roads, traffic densities, NO, NO_2_	7–11 years	2,143	Western, US	Cross-sectional/adjusted	Breath collection technique (offline and online), geocoding distance from residence to roads, road class, and density data, NO_2_ sampling/modeling^c^, questionnaire, body mass index,	Length of roads positively was associated with FeNO, with significant associations in small buffers: 46.7% [95% CI, 14.3–88.4] higher FeNO for increases in the length of all roads in 50 m buffers. Limit: rely on parent report for medication use as a confounding factor

*Asthma-related symptom and care utilization*							
Newman et al., 2014	TRAP	1–16 years	758	Central, US	Cohort/adjusted	Administrative data (ICD-9-CM) for hospital readmission (primary or secondary diagnosis), questionnaires, serum sample, allergen-specific IgE testing	Higher TRAP exposure was associated with higher readmission rate (21% versus 16%; *P* = 0.05), association was not significant after adjusting for covariates (aOR, 1.4; 95% CI, 0.9–2.2). Limit: sample was from one single institution

aOR: adjusted Odds Ratio; HR: Hazard Ratio; LUR: land use regression; ECAT: elemental carbon attributed to traffic. *Note*. (a) TRAP exposure estimated using a qualitative proximity model and quantitative LUR model; (b) modeled annual concentration estimates based on surrounding area characteristics (c) used several models including line source dispersion and regression models to map estimates.

**Table 2:** Association between specific pollutants and pediatric asthma.

Source/year	Outdoor variables	Age group	Sample size	Climate region	Study design	Assessment method	Findings and limits
*Asthma diagnosis and symptom*							
Akinbami et al., 2010	SO_2_, NO, O_3_, PM_2.5_, PM_10_	3–17 years	34,073	National, US	Cross-sectional/adjusted	National Health Interview Survey (NHIS) database; stratified multistage sampling	aORs for current asthma for the highest quartile of estimated ozone exposure: 1.56 (95% CI: 1.15, 2.10) and for recent asthma attack 1.38 (95% CI: 0.99, 1.91). Limit: county-level 12-month averages of pollution are imprecise measures of children's exposure to pollution.

Berhane et al., 2014	NO_2_, PM_10_, PM_2.5_, O_3_	5–7 years	1,211	Western, US	Cohort/adjusted	Questionnaire, FeNO measurement, ambient air monitoring stations	Increases in annual concentrations of 24-hr average NO_2_ and PM2.5 were associated with increase in FeNO. Limit: lack of information on time-activity patterns for the subjects could lead to misclassification of exposure

Cornell et al., 2012	BC, PM_2.5_		240	Northeast, US	Cross-sectional/adjusted	FeNO test, portable air sampling units, fixed BC monitor, PFT, Serum IgE	BC higher in high-asthma neighborhoods (1.59 *µ*g/m^3^ [95% CI 1.45–1.73]) than in low-asthma neighborhoods (1.16 *µ*g/m^3^ [1.06–1.27]) with *P* < 0.001. Limit: sample was limited to middle-income households

Ebisu et al., 2011	Urban land use, TRAP modeling, NO_2_	0-1 years	680	Northeast, US	Cross-sectional/adjusted	Interview, asthma diary	10% increase in urban land-use within 1,540 m buffer of infant's residence associated with 1.09-fold increase in wheeze severity. This link becames insignificant with TRAP modeling proxy^a^ added. Limit: NO2 as an indicator of overall TRAP misses other pollutants

Habre et al., 2014	PM_2.5_, O_3_,	6–14 years	36	Northeast, US	Cohort/adjusted	Symptoms diary, skin test, air sampling, air monitoring, temperature, and humidity	2 of the 3 highest frequency reactions were for ragweed (48%) and birch (39%). Exposure to O3 and particular matters was significantly associated with severe wheezing. Limit: reliance on central-site ambient measurements to assign outdoor exposure category

Jerschow, 2015	Dichlorophenols (pesticide)	≥6 years	2,125 sample (% of children unclear)	National	Cross-sectional/adjusted	NHANES, dichlorophenols measured in urine	Higher dichlorophenol levels were linked with asthma diagnosis, asthma prescriptions, missing work/school, exercise-induced wheezing in atopic wheezers. No association between dichlorophenol levels and asthma morbidity in nonatopic wheezers. Limit: reliance on self-reported data about wheezing problems.

Jung et al., 2012	Polycyclic aromatic hydrocarbons (PAH)	5-6 years	354	Northeast, US	Cohort/adjusted	Questionnaires, PAH air monitoring devices, blood samples	Repeated high exposure to pyrene was associated with report of asthma. Limit: PAH exposure was assessed only by 2 repeated measures 5 to 6 years apart, which could lead to misclassification

Lewis et al., 2013	PM_10_, PM_2.5_, PM_10-2.5_ O_3_	5–12 years	298	Upper Midwest, US	Cohort/adjusted	Respiratory symptom diary, ambient air monitoring, caregiver interview	Outdoor PM_2.5_, PM_10_, and O_3_ concentrations were associated with increased odds of respiratory symptoms, particularly in children using steroid medication. Similar associations were not realized with PM10-2.5. Limit: measuring symptoms using handwritten diaries by caregiver and the child could lead to errors.

Miller et al., 2010	Polycyclic aromatic hydrocarbons (PAH)	≥5 years	222	Northeast, US	Cohort/adjusted	Questionnaires, urine testing, immunoglobulintesting	Widely varying levels of 10 PAH urinary metabolites were detected in all children. Levels of PAH metabolites were not associated with respiratory symptoms. Limit: the half-lives of PAH metabolites are short and thus variations in exposure across time may be large.

Nishimura et al., 2013	O_3_, NO_2_, SO_2_, PM_10_, PM_2.5_	8–20 years	4,320	South, Northeast, West, Upper Midwest, Puerto Rico, US	Cohort/adjusted	Questionnaires, regional ambient air pollution data,	Early life exposure to NO_2_ was associated with risk for asthma [OR = 1.17; 95% CI 1.04–1.31] in Latino and African American children across 5 US regions. Other pollutants' impact varied across regions. Limit: measurement of PM2.5 was less complete than that of other pollutants, leading to a smaller sample.

Padula et al., 2015	PAH	9–18 years	467	West, US	Cross-sectional/adjusted	PFTs, spirometry, skin testing, fixed air monitoring, wind and humidity	Significant association between PAH and lung function testing in nonasthmatic children: increase in PAH456 was associated with decrease in FEV_1_. Limit: change in pulmonary function over time wasn't assessed

Patel et al., 2013	O_3_, PM_10_, PM_2.5_, NO_2_, PM_10-2.5_, BC	14–19 years	36	Northeast, US	Cross-sectional/adjusted	Aethalometers to measure BC, EPA systems database, R-Tube, immunosorbent assays	BC and NO2 were positively associated with airway inflammation and oxidative stress. Limit: the use of central-site PM2.5 and O3 measurements could bias the effect estimate from them toward null.

Perez et al., 2012	NO_2_, O_3_	<18 years	2.54M +	Western, US	Cross-sectional/adjusted	ACS, local surveys, EPA air quality system, ambient air monitors, proximity to traffic	8% of asthma cases were partially caused by resident proximity to major road. Link between proximity to major road and asthma exacerbations is positive. Limit: traffic density and vehicular emissions are not reflected in this metric of traffic proximity

Pongracic et al., 2010	Fungal allergen exposure	5–11 years	936 children (moderate-severe asthma)	National, US	Cohort/Adjusted for covariates	Interviews, portable air sampling, site inspections, dust samples	Excess symptom days per 2 weeks associated with increase in outdoor fungi level; increases in total fungal exposure was associated with increases in symptom days and asthma-related unscheduled visits. Limit: the study did not have children not sensitized to fungal allergens

Ratnapradipa et al., 2013	Soot, exhaust, wood or oil smoke	<5-6 (pre-school)	691	Northeast, US	Cross-sectional/adjusted	Structured interviews	Exposure to soot, exhaust, wood, or oil smoke was associated with higher risk of asthma than those never exposed. Limit: the cross-sectional nature of the study and the recall bias were associated with interview-based data

Sarnat et al., 2012	BC, PM, PM_10-2.5_, PM_2.5_,	6–12 years	58	South, US, Mexico	Cross-sectional/adjusted	eNO testing, air monitoring, air monitors, passive badge samplers, BMI measurement	There exists significant link between eNO and measures of PM and BC. PM pollutant levels predict acute respiratory responses better than NO_2_ measurements. Limit: clinical significance of the estimated increases in eNO with pollutant levels as observed here is unclear.

Spira-Cohen et al., 2011	PM_2.5_, SO_2_, Elemental carbon (EC)	10–12 years	40	Northeast, US	Cohort/adjusted	Questionnaires, air monitoring, time-activity daily diary, aethalometer, spirometry	Elevated risk of wheeze, shortness of breath, and total symptoms were associated with same-day increased personal EC, but not with personal PM2.5 mass. No associations with school-site PM2.5 or, SO_2_. Limit: a small sample size of only 40 study participant

Vette et al., 2013	PM_2.5_, BC, NO_2_, NO_x_, CO, PM_course_, VOCs	14–16 years	139	Midwest, US	Cohort/adjusted	FeNO testing, nasal lavage, F2-isoprostances, air monitoring, diaries, air monitoring	This paper is a protocol, yet preliminary data provide evidence of roadway impacts on the measured concentrations and indicate that variations in exposures between study participants are evident. Limit: full detailed results are yet to come, not in this paper

Zora et al., 2013	PM_10-2.5_, PM_2.5_, PM_10_, markers for TRAP (BC, NO_2_)	6–11 years	36	South, US	Cross-sectional/adjusted	Questionnaire, ambient air monitoring, meteorology data, pulmonary function testing	Positive (but not statistically significant) association between asthma and each single pollutant. Limit: use the questionnaire-based data as outcome variable could bring in recall bias, social desirability bias, etc.

*Asthma cost*							
Brandt et al., 2012	NO2, O3	0–17 years	1,290	Western, US	Cross-sectional/adjusted	MEPS, CHIS, NHTS, HCUP, published averages of NO2 and O3	Nearly 50% is due to regional air pollution-attributable exacerbations among children with asthma. Limit: costs are usually difficult to measure

*Asthma-related symptoms and care utilization*							
Strickland et al., 2010	PM_10-2.5_, O_3_, NO_2_, SO_2_, CO, as markers for TRAP	5–17 years	91,386	Southeast, US	Cross-sectional/adjusted	Administrative data (ICD-9) from ED visits, ambient air quality monitors, pollen counts	Asthma ED visits associated with O_3_ during warm season and cold season (Nov–Apr), several TRAP measures in warm season, PM_2.5_ and SO_2_ in warm season, PM_10-2.5_ in cold season; associations with ED visits present at relatively low ambient concentrations of studied variables. Limit: difficult to draw causal inference from cross-sectional design

Tse et al., 2015	Wildfire exposure		2,195, 3,965	West, US	Cross-sectional/adjusted	Short-acting *β*-agonist (SABA) use in obese children	SABA use increased (+16%, *P* < 0.05) in obese children (BMI > 30) compared to nonobese (BMI < 30) in 2003; increased but nonsignificant difference (+10.5%, N.S.) in SABA use in 2007. Limit: asthmatic patients may have taken preventive action to minimize the exposure

Lemke et al., 2014	NO_2_, SO_2_, VOC, PM_10_, PAH	5–89 years	2,900	Upper Midwest, US & Canada	Cross-sectional/adjusted	Geospatial data, air sampling station data, ICD-9 codes with ED visits and hospitalizations	Intraurban air quality variations related to adverse respiratory events; NO_2_, PM_10_, and VOC positively correlated with ED visits. Limit: relatively coarse temporal resolution in study design compromises generalizability

Evans et al., 2014	PM_2.5_, CO, SO_2_, O_3_	3–10 years	74	Northeast, US	Cross-sectional/adjusted	Physician visits, ER visits	Increases in UFP and CO concentration were associated with pediatric asthma visits. Increases in O_3_ were associated with less asthma visits. No associations for mode particles, BC, fine particles, or SO_2_. Limit: the monitoring station is located on a diesel bus route, which could lead to higher measured pollutant concentrations than the actual exposure among some of the study subjects.

Delfino et al., 2014	CO, N0_*x*_, PM_2.5_, O_3_, as markers for TRAP	0–18 years	11,390 visits/7,492 patients	Western, US	Cross-sectional/adjusted	Emergency Department visits, inpatient admissions; ambient air station data	ED visits and admissions for asthma were positively associated with ambient air pollution (i.e., O_3_, PM_2.5_) during the warm season, and CO, NO_2_, PM_2.5_ in the cool season. Limit: insurance status is the only individual-level sociodemographic information

BC: black carbon; ED/ER: emergency department/emergency room; eNO: exhaled nitric oxide; SABA: Short-Acting Beta-Agonists; UFP: ultrafine particles; VOC: volatile organic compound. *Note*. (a) estimated exposure levels using LUR modeling.

**Table 3:** Association between pediatric asthma and aeroallergens and other exposures.

Source/year	Outdoor variables	Age group	Sample size	Region	Study design	Assessment method	Findings and limits
*Asthma symptoms*							
Dellavalle et al., 2012	Tree, grass, weed, and all-type pollen	4–12 years	430	Northeast, US	Cross-sectional/adjusted for covariates	Questionnaire, daily diary, allergen-specific IgE panel for grass and ragweed; pollen and exposure modeling^a^	Weed pollen at low levels (6–9 grains/m3) was associated with shortness of breath, chest tightness, rescue medication use, wheeze, and persistent cough; grass pollen (≥2 grains/m3) was associated with wheeze, night symptoms, shortness of breath, and persistent cough. Limit: the study did not investigate the effect of tree pollen on sensitized children

*Asthma-related symptoms and care utlization*							
Jariwala et al., 2011	Tree pollen, ragweed, mugwort	0–18 years; and adults	52 (weekly mean ED visits)	Northeast, US	Cross-sectional/adjusted for covariates	ED visit data (ICD-9-CM codes), hospitalization data, pollen count (particles per cubic meter)	ED visits highly correlated with tree pollen (*r* = 0.90, *P* = 0.03) during Spring (March–May). No statistical association of pollen (i.e., ragweed, mugwort) during summer or fall. Limit: data limited to seven major hospitals in New York City, borough of the Bronx.

*Asthma sensitivity tests*							
Sheehan et al., 2010	Trees (birch, oak, maple, elm), grass, ragweed mix,	0–21 years	1,394	Northeast, US	Cross-sectional/adjusted for covariates	Skin prick testing database	Grass and ragweed were least common sensitizers in younger children, with rates of 1.0% (0–2 years) and 2.8% (2–4 years) for grass and 1.0% (0–2 years) and 5.7% (2–4 years) for ragweed. The rates were higher among those aged 10–12 with rates of 28.8% for grass and 34.2% for ragweed. Trees were common outdoor exposure sensitizers in all age groups. Limit: given the retrospective not all patients received the same testing

NHANES: National Health and Nutrition Examination Survey. *Note*. (a) used modeling to estimate ambient pollen exposure.
